# Randomised Controlled Clinical Trial Investigating The Impact of Implementation Planning on Behaviour Related to The Diet

**DOI:** 10.1038/s41598-018-26418-0

**Published:** 2018-05-23

**Authors:** S. O’Toole, T. Newton, R. Moazzez, A. Hasan, D. Bartlett

**Affiliations:** 1 Department of Prosthodontics, King’s College London Dental Institute, London, UK; 2 Department of Behavioural and Population Sciences, King’s College London Dental Institute, London, UK; 3 Department of Mucosal and Salivary Biology, King’s College London Dental Institute, London, UK; 4 Department of Biostatistics, King’s College London Dental Institute, London, UK

## Abstract

There is a perceived gap between dietary advice given by health practitioners and adherence to the advice by patients. We investigated whether a behaviour change technique (implementation-planning) was more effective than standard-of-care diet advice at reducing dietary acid intake using quantitative erosive tooth wear progression as an objective clinical outcome. This study was a randomised controlled, double-blind, single-centre clinical trial in the UK. Participants (n = 60) with high dietary acid intake (≥2 daily), were recruited and randomly assigned (1:1) to receive either implementation-planning or standard-of-care diet advice in a single clinical session. Questionnaires and impressions were taken at baseline and 6 months later. Dental casts were scanned using laser profilometry and superimposed using surface-matching software. Data were analysed per protocol and intention-to-treat using independent t-tests and Mann-Whitney tests. The intervention group reduced their dietary acid intake between meals to 1 intake per day compared to 2 intakes per day for the controls and demonstrated reduced dental hard tissue volume loss (−0.00 mm^3^ (SD = 0.01)) compared to controls (−0.07 mm^3^ (SD 0.17)), p = 0.049. This paper supports the use of implementation planning in clinical practice and presents a non-invasive method of intervention assessment in behaviour change. Larger trials are needed to confirm the generalisability of results.

## Introduction

A large proportion of medical and dental diseases are preventable and related to individual’s behaviours^1^. However, offering advice and patient education has had limited success in reducing disease prevalence in both medical^2^ and dental fields^3^. Increasingly, interventions based on psychological theory have been utilised to bridge the gap between a desired outcome and adherence to the behaviour necessary to achieve the outcome^4^. Gollwitzer and Sheeran distinguished between goal intentions and implementation intentions^5^. Goal intentions are deciding on the desired outcome and are linked to motivation to make the change. However, intentions have been observed to have a relatively small impact on behaviour. According to several theories such as the theory of planned behaviour, social cognitive theory and more recently the COM-B model, intentions can convert to behaviour change only if the person has the capability and opportunity to make that change^4^. Implementation planning is “the process of planning when, where and how the person will instigate responses that promote goal realization” while anticipating obstacles^5^.

Within a dental setting, planning interventions have been successful regardless of the motivation of the patient^6^. A meta-analysis of 94 papers investigating implementation planning as an intervention observed a medium to large effect (Cohen’s d = 0.65) on achieving goals^5^.This method has been used to increase adherence to flossing, resulting in reduced plaque and bleeding scores compared to flossing advice alone^7,8^. These interventions have been successful regardless of the motivation of the patient^6^. However, there is insufficient evidence to recommend routine provision of behaviour change interventions due to a lack of rigorous clinical trials^9^. Multiple reviews have called for higher quality research investigating behavioural interventions^3,9–12^, particularly with objective clinical outcomes^3,10,13^.

The ability to influence behaviours associated with the diet is of particular interest in today’s society with the increasing incidence of obesity^14^, type II diabetes^15^ and cardiovascular disease^16^. However diet-related behavioural change interventions often rely on self-reported data or invasive testing^17^. Blood tests or weight measurements are also prone to fluctuations^18^. However, the diet can have immediate, irreversible effects on the teeth.

Erosive tooth wear is defined as the chemical-mechanical dissolution of dental hard tissues by acids of non-bacterial origin^19^. This can result in dental hypersensitivity, poor aesthetics and loss of dental function^20^. Severe erosive tooth wear reduces the lifespan of teeth, significantly impacts upon quality of life^20,21^ and is increasing in prevalence^22^. A trans-European study on over 3,000 adults observed the prevalence of tooth wear to be 29%^23^. Recently, increased frequency of daily dietary acid consumption, such as acidic drinks and fruits between meals, has been associated with severe erosive tooth wear^24^. However, it is unknown if a change in behaviour, reducing the frequency of acid intake between meals, will prevent tooth wear progressing. To address this evidence gap we compared standard of care dietary advice and implementation planning as interventions in erosive tooth wear progression over a 6-month period. The null hypotheses are that implementation planning will not affect erosive tooth wear progression over 6 months and implementation planning will not reduce frequency of dietary acid intake compared to standard of care dietary advice.

## Methods

This study was a randomised controlled, parallel group, double-blind, single-centre clinical trial completed in Guy’s Dental Hospital, London, UK. The study protocol was approved by Nottingham National Health Research Authority East Midlands (Reference 14/EM/1171) and adheres to the Consolidated Standards of Reporting Trials (CONSORT) guidelines^25^. This trial was first registered under clinicaltrials.gov (registration ID: NCT02493803) on the 18^th^ May 2015. All participants provided fully-informed, written consent and the trial is now closed.

### Participants

The source population were participants referred via their general dental practitioner for assessment of moderate-to-severe erosive tooth wear at Guy’s Dental Hospital between 27^th^ October 2014 and 10^th^ February 2016. Males and females, aged 25–70 years, were screened and those conforming to the inclusion and exclusion criteria invited to participate. Following written consent, current dietary behaviour was recorded using a previously validated interviewer-led, oral questionnaire^24^. This questionnaire contained 9 items and recorded the daily frequency, timing (with meals or between meals), duration of consumption of fruits, fruit drinks, carbonated beverages and other acidic drinks. In addition, the type of holder (cup, glass, bottle, can), presence of sipping, swishing or holding drinks prior to swallowing were recorded.

The Basic Erosive Wear Examination (BEWE) score^26^ was assessed with the patient in a reclined position in a dental chair using good lighting, teeth were dried with compressed air and the buccal, occlusal and palatal/lingual surfaces examined for each tooth, excluding third molars, without magnification. The inclusion criteria were: a minimum of 20 teeth (10 in each jaw) with a BEWE cumulative score greater than or equal to 8 but with at least one score of 3 on the index teeth (the occlusal surfaces of the lower molars or the buccal/palatal surface of the upper central incisors), a high acidic diet (at least two daily incidences of dietary acid intake) and ability to provide written consent to the study. Participants were excluded if they had orthodontic appliances, dentine hypersensitivity, missing anterior teeth, anterior crowns/bridges or cavitated caries on more than one tooth. A history of eating disorders, gastro-oesophageal reflux, xerostomia, bruxism, requiring antibiotic pre-medication prior to dental treatment, current pregnancy or recent/concurrent participation in another clinical study also excluded the participant from this trial.

Participants were allocated a unique trial identifier number based upon sequence of recruitment. Simple Random Sampling (SRS) using Excel (Microsoft Office Excel 2010, Redmond, USA), was used by a trial statistician to allocate the patient to either the intervention or standard care group. Simple randomisation was used as, to date, there are no known demographic predictors of tooth wear progression. The participant was blinded to whether they received standard of care diet advice or implementation planning. The clinical investigator was not blinded to the intervention but was blinded during the analysis process and outcome measurement.

### Interventions

A single research dentist was trained how to use the intervention scripts and delivered both interventions. Participants in the intervention group were asked to identify and write down the behaviour they wished to change and the substitution/abstention they were going to make. Participants were prompted to identify obstacles that would prevent them from making their chosen substitution/abstention, how they might overcome these obstacles and write down plans that would help them to make the change (often called an if-then plan). A prompt-sheet with a list of dietary acids and healthy substitutions with low erosive potential was provided (Appendix 1). Participants were encouraged to keep the plan in a place that would be visible daily at work or at home (Appendix 1). Researcher scripts and guidelines, with different dialogues depending on the risk behaviour, were formulated to ensure dietary interventions were standardised and are presented in Appendix 2. This intervention lasted 3–5 minutes, less than the time originally hypothesised in the trial protocol.

For the control group (standard-of-care), the following statement was said: *“Our examination has revealed that you show signs of erosive damage on your teeth. This is most likely to be due to a combination of the foods and drinks that you choose, when you have them and when you brush your teeth. We recommend that you cut down on the frequency of having acidic foods and drink.”* This intervention lasted less than 1 minute.

Scavenger alginate impressions (Alginate Plus, DF Fast Set, Henry Schein, Kent, UK) were taken of the upper and lower arches to remove debris. The index teeth were isolated with cotton wool rolls, dried with compressed air and addition-cured silicone impressions taken (Extrude, Kerr Dental, Peterborough, UK). The impressions were disinfected by immersing in Perform ID (Schülke & Mayr UK Ltd. Sheffield, UK) for 10 minutes and left to rest undisturbed for 24 hours before being poured in type 4 dental stone following the manufacturers recommendations (Fujirock EP, premium line pastel yellow, GC United Kingdom Ltd., Newport Pagnell, UK). After a minimum of 24 hours, the index surfaces were scanned using a triangulation laser profilometer (Xyris 2000TL. TaiCaan, Southampton, UK) and data recorded every 50 µm, scanning from left to right in a raster pattern, at medium precision mode (scanning speed of 2.81 mm/s)^27^. This laser is accurate to 1.3 µm and repeatable to 1.6 µm. The coefficient of variation on volume measurements is <5%^28^.

Participants were then invited to return 6 months (±7 days) later (T1) and the dietary questionnaire repeated. New impressions were taken of the index teeth using the same impression methods reported above. All scanning, superimposition and data analysis were performed blinded to the group allocation. Sequential 3D scans obtained at T0 and T1 were aligned by minimising the root mean square difference between data points using Geomagic Control software (3D systems, Darmstadt, Germany). A standardised representative area, 6 × 4 mm, was conveniently selected from the central part of the digitised surface. The centre was arbitrarily defined at the intersection of the mesio-distal and bucco-lingual length for molars and at the intersection of the cervico-incisal and mesio-distal length for incisors. The volume difference between the two scans was calculated by determining the difference between the scanned surface and a defined plane at T1 from the baseline at T0.

### Outcomes

The primary self-reported outcome was frequency of dietary acid intake between meals extracted from the diet questionnaires obtained at T0 and T1. The daily frequency of acid intake (as an integer) was estimated as the number of times fruit (apples, citrus, grapes, berries and any other fruit) and/or acidic drinks (carbonated drinks, fruit drinks, any other acidic drinks e.g. fruit teas, wine) were reported to be consumed both between meals and the total number of times per day. This was recorded at T0 and T1 for each participant to give a pre- and post-intervention assessment score. The primary clinical outcome was quantitative dental hard tissue volume loss over the 6-month period. This was the mean volume hard tissue loss for all surfaces analysed (the buccal and palatal surfaces of the central incisors and the occlusal surfaces of the lower first molars) which epidemiologically are most likely to be associated with tooth wear.

### Statistical methods

Previous work within our group observed differences of 15 µm at surface level between participants with high and low levels of wear progression^27^, yielding an effect size of 0.78. To detect a difference in wear with 80% power and at 5% significance level while anticipating a 10% drop out rate, a total sample size of 60 participants, to ensure 27 participants completed the study in each group, were recruited.

The datasets generated and analysed during the current study are available from the corresponding author on reasonable request. All analyses were performed in the Statistical Package for Social Sciences version 23 for Windows (IBM Corporation, Armonk, New York, USA). Data were checked for normality using histograms, boxplots and the Shapiro-Wilk test.

The frequency of dietary acid intake data was not normally distributed. Independent samples Mann-Whitney U tests between groups at baseline and post intervention were performed both per-protocol and intention-to-treat analysis after missing values were imputed by multiple imputation.

The correlation between the volume changes detected on the different surfaces was analysed. Since there was evidence for a clustering effect of the surfaces within patients (as reflected by an intraclass correlation coefficient of 0.92), the mean of all analysed surfaces per patient was used to detect differences between groups. Data at patient level was normally distributed, therefore differences between groups were assessed using independent t-tests per protocol and intention-to-treat analysis after missing values were imputed by multiple imputation. To estimate if any of the surfaces were associated with tooth wear progression for the study population, a fixed-effects multi-level model was used with the mean volume loss for each individual surface type per patient as the dependent variable, mean volume loss per patient as the repeated measure, gender as a factor and age as a covariate. Significance was set at p < 0.05.

## Results

### Participant and Data Overview

A total of 98 identified participants were assessed for eligibility. Of those, 38 were excluded as they did not meet the full inclusion criteria (n = 33), declined to participate (n = 3) and other reasons (n = 2). Remaining participants (n = 60) were randomly allocated to either group. One participant from each group was lost to follow up as s/he was not in the country at the time of recall. In addition, one participant from the control group was lost to a fatal accident unrelated to the trial. This resulted in a total of 28 participants in the control group and 29 participants in the intervention group. Table 1 reports the patient demographics between the two groups with no statistical differences observed in age (p = 0.529), gender (p = 0.116). or baseline total BEWE score (p = 0.769). The mean BEWE score for the standard-of-care group at T0 was 14.7 (SD 2.5) and 14.9 (SD 2.3) at T1 and for the intervention group was 14.8 (SD 2.2) at T0 and 14.8 (SD 2.2) at T1 and there was no statistical difference in scores between the groups at T0 (0.769) or at T1 (p = 0.849). (Insert Table 1).

**Table 1:** Participant Demographics for each Group.

	Standard of Care (n = 28)		Behaviour Change Intervention (n = 29)	
	n	%	n	%
Age				
18–25	4	14.3	4	13.8
26–35	11	39.3	13	44.8
36–45	5	17.9	5	17.2
46–55	4	14.3	6	20.7
56–65	4	14.3	1	3.4
Gender				
Male	11	39.3	17	58.6
Female	17	60.7	12	41.4
	**Mean**	**SD**	**Mean**	**SD**
BEWE visit 1	14.7	(2.5)	14.8	(2.2)
BEWE visit 2	14.9	(2.3)	14.8	(2.2)

There were no dietary differences between groups at T0 for both the frequency of dietary acid intake between meals (median intake: 3 IQR 2.0,4.0 for both groups, p = 0.783) and the frequency of daily dietary acids (median intake: 4.0, IQR 2.0,5.8 for the control group and median 4.0, IQR 2.0,5.5 for the control, p = 0.619). Both groups reduced their frequency of dietary acids over the trial period (p < 0.001). At T1, the control group reduced their daily dietary acid consumption to a median of 2.5 intakes (IQR 1.0,4.0, Range 0–6) and the intervention group reduced their daily dietary acid consumption to a median of 1.0 intake (IQR 0.5, 2.0), Range 0–4) and this difference was statistically significant (p = 0.002). Between meals, the control group reduced their daily frequency of dietary acid consumption to a median of 1.0 intake (IQR 1.0, 3.0, Range: 0–4) and the intervention group had reduced to a median of 1 (IQR 0, 1, Range 0–4) and this difference was statistically significant (p = 0.003). All changes in self-reported frequency of dietary acid intake are reported in Table 2.

**Table 2:** Self-reported frequency of dietary acid intake.

	Standard of care		Behaviour Intervention		p value between groups per protocol	p value between groups intention-to-treat
	Median (IQR)	Mean (SD)	Median (IQR)	Mean (SD)		
**Daily frequency of dietary acid intake between meals**						
At baseline (T0)	3.0 (2.0,4.0)	3.1 (1.8)	3.0 (2.0,4.0)	1.75 (1.3)	0.783	
6 months (T1)	1.0 (1.0,3.0)	1.8 (1.3)	1.0 (0.0,1.0)	0.8 (1.0)	0.002**	0.002**
p value within group	<0.001*		<0.001*			
**Daily frequency of dietary acid intake**						
At baseline (T0)	4.0 (2.0,5.8)	2.6 (1.6)	4.0 (2.0,5.5)	3.8 (1.5)	0.619	
6 months (T1)	2.5 (1.0,4.0)	1.0 (2.3)	1.0 (0.5,2.0)	1.0 (1.2)	0.003**	0.004**
p value within group	<0.001*		<0.001*			

^*^Statistically significant.

Of the possible 342 tooth surface pairs, which were scanned and superimposed in both groups, 48 were excluded. The reasons for exclusion were restoration of the surface (n = 15), fracture of the surface (n = 15) and failure of accurate data point alignment (n = 18). There were no statistically significant differences between the numbers of surface types included for analysis in each group. A flow chart for the data collection for the intervention and control group is reported in Appendix 3 according to Consolidated Standards of Reporting Trials (CONSORT) guidelines^25^.

The mean volume loss per surface for each patient was −0.07 mm^3^ (SD 0.17) for the control group and −0.00 mm^3^ (SD 0.01) per surface for the intervention group. This difference was statistically significant (p = 0.049) when analysed per protocol and with intention-to treat. Results are reported in Table 3. The buccal surfaces of upper central incisors and palatal surface of UL1 were significantly associated with mean volume loss (all p < 0.05) whilst adjusting for clustering of surfaces within patients, the intervention, gender and age.

**Table 3:** Dental Hard Tissue Loss Measured by Volume change analysis (mm^3^) During the 6 Month Trial Period.

	Standard of care		Behaviour Intervention		p value Per Protocol	p value Intention To Treat
	Median (IQR)	Mean (SD)	Median (IQR)	Mean (SD)		
	n = 28		n = 29			
Mean Volume Loss Per Surface Per Patient	−0.09 (−0.14, 0.05)	−0.07 (0.17)	−0.02 (−0.08, 0.09)	0.01 (0.13)	0.049*	0.049*

## Discussion

Within the limitations of this single-centre, hospital-based trial, the implementation planning intervention used demonstrated a positive effect. Those who performed implementation planning significantly reduced their self-reported dietary acid intake between meals. The intervention group also had significantly reduced dental hard tissue volume loss compared to those receiving standard-of-care. It is also worth considering that the clinical differences observed between groups were at the borderline level of significance. Previous studies on low risk adults have monitored patients for 2 year periods^29^ thus the time period in this study resulted in reduced clinical progression. However, considering these limitations, this intervention supports the use of behavioural change interventions in clinical practice to improve clinical outcomes^6–8^. Motivational interviewing has also been attempted in practice settings and there is evidence demonstrating success^30^. However, motivational interviewing techniques require intensive training and are reliant upon a good repertoire between the patient and the clinician^30^. If-then planning may have additional benefits over other forms of behaviour change interventions within an assiduous clinical practice. It is brief and directive, requiring no prior training of the health provider and can be done without establishing an ideal long-term collaborative relationship^5^. The clinician and the patient quickly identify which behaviour they are going to target, make specific plans to target the behaviour, discuss obstacles to the specific behaviour change and plans to overcome these obstacles. Planning actions have been successful regardless of motivation levels of the patient^6^ which also contrasts with motivational interviewing techniques.

An attempt was made to address any confounding factors. The known clinical confounding factors of xerostomia, dental parafunction or intrinsic acid damage were excluded. The randomisation process successfully balanced the groups with no statistical differences between ages, gender, numbers of surfaces analysed and baseline frequency of dietary acid intake. The baseline BEWE scores were not different between groups, showing similar levels of tooth wear at inclusion. Both the participants and the statistician performing analysis were blinded to the allocation group. However, self-reported outcome measures are subject to reporting bias. Participants may have under-reported dietary acid intake due to social desirability bias. Patients recruited from a hospital population may be more aware of their condition and subsequently more motivated to change their behaviour. Furthermore, customisation of advice has also been reported to offer improved outcomes over generalised advice in other fields of diet change^31,32^. Having stated these limitations, one could argue that the information provided to the control group by the detailed dietary questionnaire was also customised. It is also due to this bias that we wished to also measure an objective clinical outcome. The intervention group demonstrated reduced tooth wear progression compared to the control group and it is promising that the clinical outcomes reflect the self-reported behavioural outcomes.

The clinical impact of this intervention can best be assessed by comparing it to previously reported wear rates of a mean volumetric loss per surface of 0.04 mm^3^ per annum^29^. Our high-risk standard of care group showed wear rates over 3 times greater than this (0.07 mm^3^ over 6 months) and represents a loss of 1% of the tooth surface per year. In contrast, those who performed implementation planning demonstrated wear rates sizeably less (0.01 mm^3^ over 6 months) than the reported norm. This simple behaviour change intervention warrants further investigation and suggests digital monitoring of erosive tooth wear as an effective, objective, non-invasive and rapid clinical outcome with which to measure preventive interventions. The buccal surfaces of the central incisors were the surfaces most highly correlated with wear progression for all patients in this trial and have also been associated with dietary sources of acid epidemiologically^33,34^. They are often the first surface to contact a fluid and they have also been observed to maintain a low pH for longer after dietary acid consumption^35^. The ability for improved visualisation and isolation in the anterior teeth, in addition to more favourable topography of the smoother surfaces creates less noise in the data. For future intervention assessment, volume change measurement could be limited to the buccal surfaces of the central incisors to increase trial efficiency.

This research supports the use of implementation planning to help achieve behaviour change in clinical practice. This study also presents a novel method of intervention assessment in behaviour change. However, this was a single-centre trial, performed by a single operator with clinical differences at the borderline level of significance. To assess the generalisability of the results, the trial should be replicated in the general population with multiple health care professionals. Further trials, with an increased sample size and over a longer time period are needed to fully assess the effectiveness of the intervention, adherence to the behaviour change and maintenance of dietary changes.

## Electronic supplementary material

Consort checklist

Intervention form and scripts, consort flow diagram
