# Supplementary material for: Randomised Controlled Clinical Trial Investigating The Impact of Implementation Planning on Behaviour Related to The Diet
Source: Sci Rep. 2018 May 23;8:8024. doi: 10.1038/s41598-018-26418-0 (PMC5966402; doi:10.1038/s41598-018-26418-0)

TITLE: RANDOMISED CONTROLLED CLINICAL TRIAL INVESTIGATING THE IMPACT OF IMPLEMENTATION PLANNING ON BEHAVIOUR RELATED TO THE DIET

O'Toole S*, Newton T, Moazzez R, Hasan A, Bartlett D.

* Corresponding Author: Dr. Saoirse O'Toole. Department of Prosthodontics, King's College London Dental Institute, saoirse.otoole@kcl.ac.uk

Prof. Jonathan Tim Newton. Department of Behavioural and Population Sciences, King's College London Dental Institute, tim.newton@kcl.ac.uk

Dr. Rebecca Moazzez, Department of Mucosal and Salivary Biology, King's College London Dental Institute, rebecca.v.moazzez@kcl.ac.uk

Dr. Adam Hasan, Department of Biostatistics, King's College London Dental Institute, adam.hasan@kcl.ac.uk

Prof. David Bartlett, Department of Prosthodontics, King's College London Dental Institute, david.bartlett@kcl.ac.uk

APPENDIX 1: IF-THEN PLAN

Making a change! Reducing the risk of acid erosion – IF/THEN planning.

What do I plan to do:

(Please tick one)

☐	I am going to reduce how often I eat things which can cause erosion to my teeth.
☐	I am going to reduce how often I drink things which can cause erosion to my teeth.
☐	I am going to brush my teeth BEFORE I eat.

What I will do instead:

What will help me to make this change:

APPENDIX 2: RESEARCHER SCRIPTS FOR DIETARY ADVICE

Script for control group

Our examination has revealed that you show signs of erosion of the teeth. This is most likely to be due to a combination of the foods and drinks that you have, when you have them and when you brush your teeth.

We recommend that you cut down on the frequency of having acidic foods and drink.

Script for intervention group

Step 1: Identifying target behaviour

Resources:

- Prompt sheet – foods that have high erosive potential: List

Script:

Our examination has revealed that you show signs of erosion of the teeth. This is most likely to be due to a combination of the foods and drinks that you have, when you have them and when you brush your teeth. Our aim is to decrease the amount of erosion that is happening, and this means helping you to change these three behaviours.

To summarise from the questionnaire:
You have X number of foods during the day where there is a possibility of acid attacks on your teeth
You have Y number of drinks that can cause erosion of the teeth
You tend to clean your teeth just after eating or drinking / You always clean your teeth before eating – which is the best way to protect your teeth from erosion.
These behaviours damaged your teeth and are the behaviours we want to change to stop the damage from getting worse. From the start can I say that we don't want you to stop eating these foods, but we recommend that instead of eating them one at a time throughout the day, you try to eat them all at once at mealtimes once or twice a day.

Step 2: Behaviour specific intervention

Resources:

- Prompt sheet – IF/THEN planning

2.1 Selecting the target

Script:

Which do you think would be easier to tackle first – the foods or the drinks? (Consider giving advice from the professional's perspective, such as "From my perspective I think we could make the biggest difference by …")

OK, you think it would be easiest to …

2.2 Target the frequency of foods with high erosive potential.	2.3 Target the frequency of drinks with high erosive potential.	2.4 Target brushing after an acidic attack
Looking at when you have your acidic foods, which one would be the easiest to stop?	Looking at when you have your drinks, which one would be the easiest to change?	You mentioned that you tend to clean your teeth after you have eaten. This causes problems because the acid on the surface of the teeth works with the brushing to wear away the teeth. Ideally you should clean your teeth before meals and not clean your teeth at least one hour after eating or drinking any of the foods on the list.
↓	↓	↓
If you stopped that snack, what would you do instead?<br><br>(Options are Substitution or Remove: Prompt for substitution of a non-erosive snack / eat snack at previous meal so feel fuller. If suggests alternative then prompt How would you remember to eat that instead? What would help you to remember? Would it mean taking something to work?<br><br>If suggests "Have nothing" prompt: How easy would that be – do you think you might find yourself getting hungrier later on? What would you do then? - Prompt for not snacking again, suggest eat more at previous meal to sustain hunger.)	If you changed that drink, what would you drink instead?<br>(Options are Substitution or Remove: Prompt for substitution of a non-erosive drink. If suggests alternative then prompt How would you remember to drink that instead? What would help you to remember? Would it mean taking something to work or school?<br><br>If suggests "Have nothing" prompt: How easy would that be – do you think you might find yourself getting really thirsty? What would you do then? - Prompt for substitution with water.)	How easy do you think it would be to change from cleaning your teeth after to before meals?<br><br>(What would be difficult about that? Prompt – feel odd, teeth might feel 'dirty', forgetting)<br><br>Yes, it would feel odd at first but after a while you will get used to it, like any new habit it feels odd at first but after a while it becomes normal. (Give example e.g. new jewelry, having a mobile 'phone – before we had them it didn't feel odd not having one, but now it does etc.)<br><br>(If concerned about mouth feel, prompt for alternatives)<br><br>You could try using mouthwash after a meal to make your mouth feel clean – this is good because it doesn't involve the brushing.
↓	↓	↓
We have found that a useful way to remember this is to make a note on this sheet. We complete it like this (Note target on prompt sheet)	We have found that a useful way to remember this is to make a note on this sheet. We complete it like this (Note target on prompt sheet)	Now there is one exception to this – when you brush your teeth last thing at night. Once you have cleaned your teeth at night you should not have anything else to eat. In this case you should try not to eat at least 1 hour before you brush your teeth.

		Which of your snacks or meals would that involve? (Refer to Recording sheet for self-reported frequency of snacking and drinking)<br><br>If you stopped that snack, what would you do instead?<br><br>(Options are Substitution or Remove: Prompt for substitution of a non-erosive snack / eat snack at previous meal so feel fuller. If suggests alternative then prompt How would you remember to eat that instead? What would help you to remember? Would it mean taking something to work or school?<br><br>If suggests “Have nothing” prompt: How easy would that be – do you think you might find yourself getting hungrier later on? What would you do then? - Prompt for not snacking again, suggest eat more at previous meal to sustain hunger.)
↓	↓	↓
Then we say what you will do instead (Note substitution or removal)	Then we say what you will do instead (Note substitution or removal)	We have found that a useful way to remember this is to make a note on this sheet. We complete it like this (Note target on prompt sheet)
↓	↓	↓
And then list all the things that will help you to make that change (Note supports already mentioned)<br><br>Can you think of anything else that will help you make this change (Prompt for – tell friends and family so they don’t offer you snacks, reward self for change, put stickers on fridge / other places).	And then list all the things that will help you to make that change (Note supports already mentioned)<br><br>Can you think of anything else that will help you make this change (Prompt for – tell friends and family so they don’t offer you snacks, reward self for change, put stickers on fridge / other places).	Then we say what you will do instead (Note substitution or removal)
		↓
		And then list all the things that will help you to make that change (Note supports already mentioned)<br><br>Can you think of anything else that will help you make this change (Prompt for – tell friends and family so they don’t offer you snacks, reward self for change, put stickers on fridge / other places).

Appendix 3: Consort flow chart

CONSORT
TRANSPARENT REPORTING of TRIALS

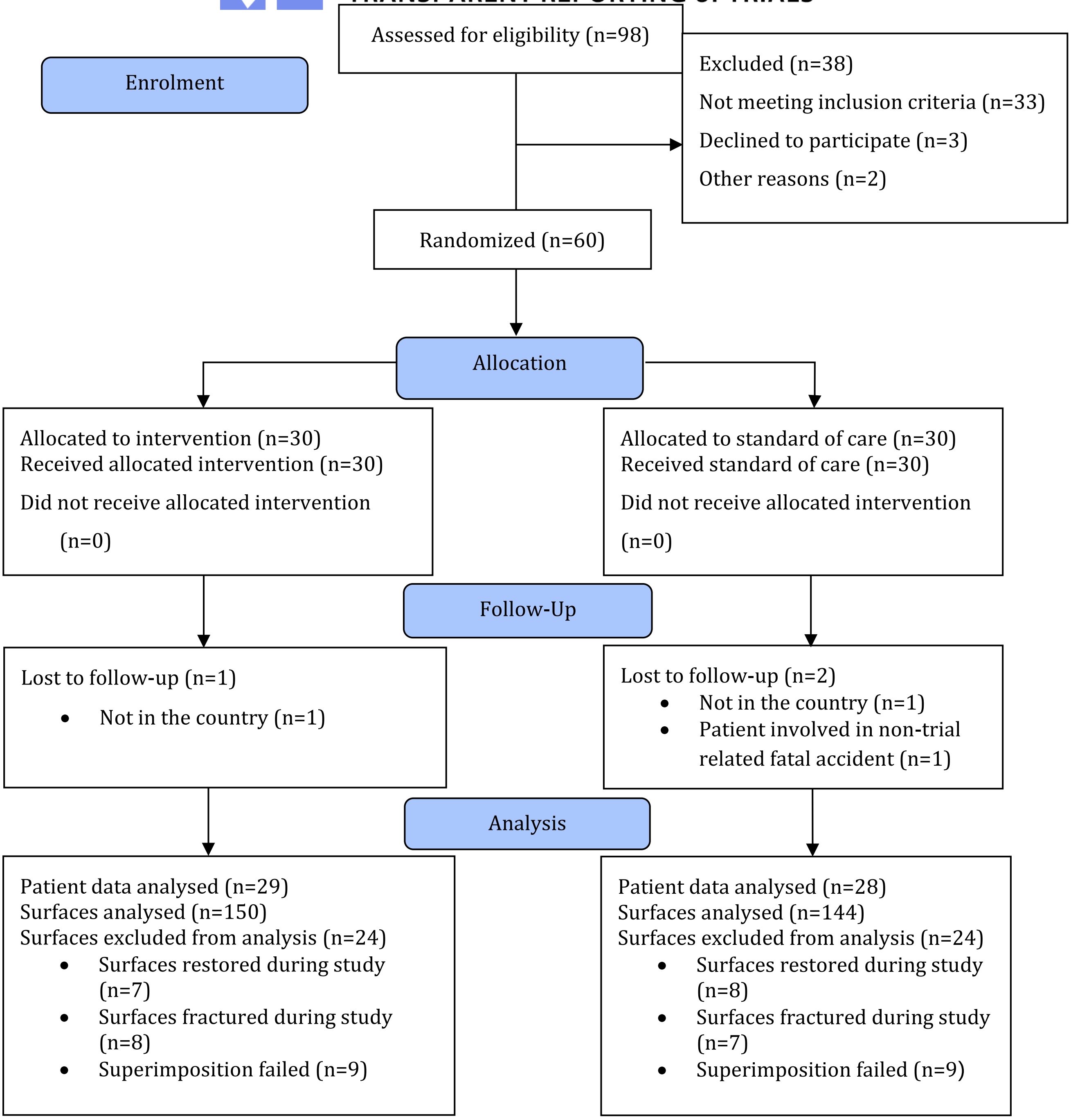

Supplement: Supplementary file 2 — Intervention form and scripts, consort flow diagram [file 41598_2018_26418_MOESM2_ESM.pdf]
